# Variable GLP-1 receptor expression across diverse neuroendocrine neoplasms: implications for incretin therapies

**DOI:** 10.1530/EO-25-0051

**Published:** 2025-11-25

**Authors:** Po Hien Ear, Sophia A Hueser, Reese E Townsend, Jessica E Maxwell, Ellen Abusada, Carlos H F Chan, Dawn E Quelle, Joseph S Dillon, James R Howe, Andrew M Bellizzi

**Affiliations:** ^1^ Department of Surgery, University of Iowa Carver College of Medicine, Iowa City, Iowa, USA; ^2^ Department of Surgical Oncology, The University of Texas MD Anderson Cancer Center, Houston, Texas, USA; ^3^ Department of Pathology University of Iowa Carver College of Medicine, Iowa City, Iowa, USA; ^4^ Department of Neuroscience and Pharmacology, University of Iowa Carver College of Medicine, Iowa City, Iowa, USA; ^5^ Department of Internal Medicine, University of Iowa Carver College of Medicine, Iowa City, Iowa, USA

**Keywords:** immunohistochemistry, insulinomas, neuroendocrine tumors, GLP-1 receptor, GLP-1R agonist

## Abstract

Neuroendocrine neoplasms (NENs) are rare cancers originating in various organs and are further classified as well-differentiated neuroendocrine tumors (NETs) and poorly differentiated neuroendocrine carcinomas (NECs). Commonly used drugs such as proton pump inhibitors can promote gastric NEN growth. Nowadays, incretin mimetic drugs such as glucagon-like peptide 1 receptor (GLP-1R) agonists have gained extensive popularity for the treatment of diabetes and obesity. These drugs target GLP-1R, and their use in neuroendocrine cancer patients with medullary thyroid carcinoma (thyroid NET) or multiple endocrine neoplasia syndrome type 2 is deemed contraindicated. Previous studies investigated GLP-1R expression in small subsets of NENs. Here, we assessed GLP-1R expression by immunohistochemistry in a large collection of 576 patient NENs from 13 sites of origin. We identified that 7% of NENs stained positive for GLP-1R. They were from five NEN types: duodenal NETs (dNETs), gastric NETs, pancreatic NETs, pheochromocytomas, and lung NETs. We then validated the dNET spheroids for response to an incretin mimetic drug and found activation of the MAPK pathway and growth. While GLP-1R agonists are contraindicated in patients with thyroid NETs, none of our 29 thyroid NETs expressed GLP-1R. Hence, in contrast to rodent studies, GLP-1R agonists may have little effect on human thyroid NETs since they do not express the receptor. Interestingly, ileal NETs also showed no expression of GLP-1R. More preclinical research examining potential oncogenic effects of incretin mimetics on the five subtypes of GLP-1R-positive NENs is needed to better understand their safety.

## Main text

**Figure 1:**
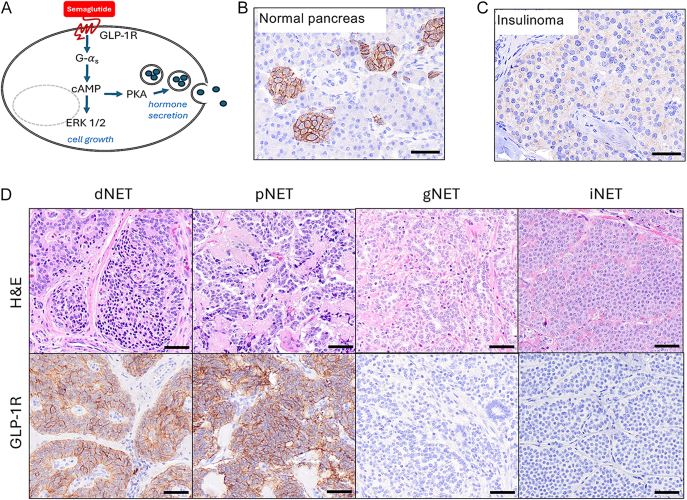
GLP-1R signaling pathways and optimization of an immunohistochemistry assay for detecting receptor expression. (A) Semaglutide and GLP-1R signaling pathways. (B) Establishment of an immunohistochemical (IHC) assay to stain GLP-1R in islets of Langerhans. (C) GLP-1R staining in an insulinoma. (D) Representative image of H&E and GLP-1R IHC staining in duodenal (dNET), pancreatic (pNET), gastric (gNET), and ileal NETs (iNET).

Neuroendocrine neoplasms (NENs) are a family of rare cancers that can occur in different parts of the body. NENs are subdivided into well-differentiated, slowly growing tumors (NETs) and more aggressive, poorly differentiated carcinomas (NECs). NENs are frequently metastatic, challenging to treat, and steadily rising in incidence and prevalence. Ubiquitous proton pump inhibitor use has recently been linked to the development of gastric NENs through the trophic effect of gastrin on gastrin receptor-expressing enterochromaffin-like cells (1, 2). Glucagon-like peptide 1 receptor agonist (GLP-1RA) incretin therapies are now used by over 12% of US adults for diabetes and weight loss (3). These agents are contraindicated in patients with thyroid NETs (i.e., medullary thyroid carcinoma) or multiple endocrine neoplasia type 2 (MEN2) based on rodent studies suggesting that GLP-1RAs promote tumor growth via activation of GLP-1R on rodent thyroid C-cells (4, 5). The risk posed by these drugs in humans remains unclear (6), and there is conflicting evidence regarding GLP-1R expression by human C-cells (4, 7, 8) and their safety (9). Induction of tumor growth by GLP-1RA drugs on receptor-expressing NENs is a valid concern. In that regard, insulinomas have been reported to express GLP-1R (10). Furthermore, we demonstrated that semaglutide, a GLP-1RA marketed as Ozempic, promotes tumor cell growth *in vitro* and *in vivo* in human small bowel and pancreatic NET GLP-1R-expressing cell lines by activating the mitogen-activated protein kinase (MAPK) pathway (Fig. 1A), but not in those that lack the receptor (11). Because NENs are relatively rare cancers, little is known about GLP-1R expression in NENs from different anatomical locations or their response to semaglutide. Here, we assessed GLP-1R expression by immunohistochemistry (IHC) in 576 patient NENs of diverse origin and determined sensitivity to semaglutide using novel patient-derived NET spheroids.

We established a clinical-grade IHC assay for GLP-1R expression and demonstrated its specificity for staining islets of Langerhans in normal human pancreas (Fig. 1B) and an insulinoma (Fig. 1C). We then investigated GLP-1R expression in 13 types of NENs using tissue microarrays (TMAs) containing 576 specimens from 350 patients (Fig. 1D and Table 1). Only five NEN types stained positively for GLP-1R, including 45% of duodenal NETs (dNETs), 17% of gastric NETs (gNETs), 13% of pancreatic NETs (pNETs), 9% of pheochromocytomas, and 2% of lung NETs (Table 1). Although 45% of dNETs stained positively for GLP-1R, the mean intensity score (H-score) was modest at 57. Pancreatic NETs displayed the highest average level of GLP-1R staining (mean H-score of 115), whereas gNETs had the lowest level (mean H-score of 4) (Fig. 1D and Table 1). The three NETs subtypes with the highest GLP-1R-positive signal were from the foregut. Somewhat surprisingly, no thyroid or ileal NETs (iNETs) stained positively for GLP-1R. These data suggest that GLP-1RAs may have unwanted oncogenic effects on receptor-positive NET subtypes (e.g., duodenal, gastric, and pancreatic NETs) and are less likely to affect NENs lacking the receptor (e.g., thyroid and ileal NETs).

**Table 1:** Quantification of GLP-1R staining in NEN tissue microarrays (TMA).

Tumor type	NEN type	Number of tumors	Number of patients	Any positive (any non-zero H-score) (%)	Mean (median) H-score if positive	Range of positive H-scores
Duodenum NET (dNET)	NET	62	50	45%	57 (38)	0.3–275
Gastric NET (gNET)	NET	12	12	17%	3 (3)	0.3–5
Pancreas NET (pNET)	NET	78	59	13%	115 (110)	5–285
Pheochromocytoma	NEN	22	22	9%	4 (4)	0.3–7
Lung NET (carcinoid)	NET	42	42	2%	20 (20)	20
Thyroid NET (medullary)	NET	29	23	0%	NA	NA
Ileal NET (iNET)	NET	252	63	0%	NA	NA
Appendix NET	NET	6	6	0%	NA	NA
Rectum NET	NET	6	6	0%	NA	NA
Paraganglioma	NEN	22	22	0%	NA	NA
Merkel cell carcinoma	NEC	23	23	0%	NA	NA
Small cell lung NEC	NEC	10	10	0%	NA	NA
Extrapulmonary visceral NEC	NEC	12	12	0%	NA	NA
T**otal**		**576**	**350**			

Since GLP-1R expression was previously reported in insulinomas, we clinically annotated and performed insulin IHC on the ten GLP-1R-positive pNETs (Supplemental Table 1 (see section on Supplementary materials given at the end of the article)). Only two tumors were clinically insulinomas, while three others were insulin IHC positive. In addition, there were five nonfunctional pNETs, two serotonin-expressing samples from one patient, and one gastrin-expressing tumor. Thus, GLP-1R-positivity is not restricted to insulinoma or insulin IHC-positive pNETs.

**Figure 2:**
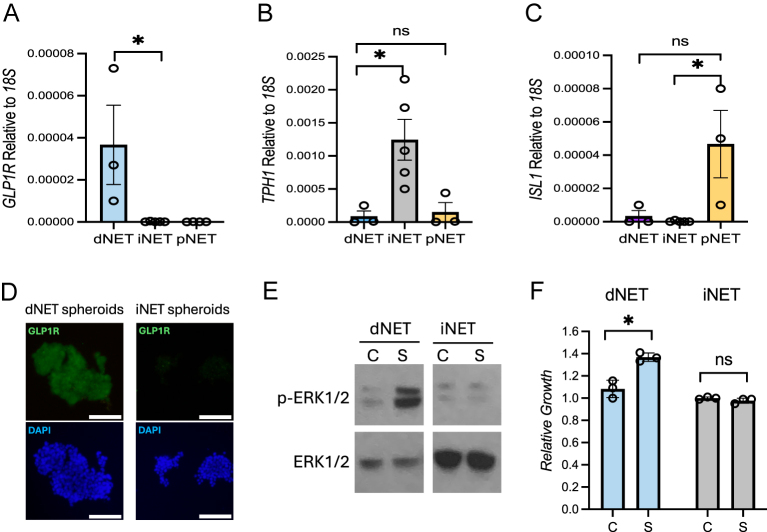
Variable GLP-1R expression depending on NEN type correlates with tumor response to GLP-1R agonist treatment. (A, B, C) qPCR for *GLP1R*, *TPH1*, and *ISL1* expression levels in patient-derived NET spheroid samples. (D) Immunofluorescence staining of patient-derived dNET spheroids for GLP-1R levels. (E) Effect of semaglutide (S) relative to the vehicle control (C) on the phosphorylation of ERK1/2 (p-ERK1/2) in dNET and iNET spheroids. (F) Growth of dNET and iNET spheroids treated with 100 nM of semaglutide (S) relative to the vehicle control (C) for 2 weeks. Scale bars represent 100 μm. *indicates *P*-value <0.05. ns represents not significant.

Patient-derived NET spheroids were generated and screened for GLP-1R expression by quantitative PCR and immunofluorescence staining. In agreement with the TMA data, dNET spheroids (*n* = 3) expressed the highest levels of *GLP-1R* mRNA relative to iNET (*n* = 5) and pNET (*n* = 3) spheroids (Fig. 2A). Ileal NET spheroids had high mRNA expression of the tryptophan hydroxylase 1 (*TPH1*) gene, and pNET spheroids had high levels of the islet-1 (*ISL1*) gene (Fig. 2B and C). The GLP-1R protein expression levels of dNET spheroids were confirmed by immunofluorescence staining (Fig. 2D). Clinical information for the parent patient tumors is provided in Supplemental Table 2. We then tested the spheroids for response to semaglutide. As predicted, semaglutide activated the MAPK pathway in GLP-1R-expressing dNET spheroids, as measured by an increase in the phosphorylation of ERK1/2, whereas receptor-negative iNET spheroids were unresponsive to the drug (Fig. 2E). In addition, semaglutide increased dNET spheroid growth by 30% after 2 weeks of treatment at 100 nM concentration (Fig. 2F). No increase in ERK1/2 phosphorylation or iNET spheroid growth was observed under the same conditions (Fig. 2E and F).

In conclusion, only 7% of 576 NENs were positive for GLP-1R, limited to five NEN subtypes. dNETs most frequently expressed the receptor (45% were positive), albeit typically at modest levels, while a smaller percentage of pNETs (13%) were GLP-1R positive, though typically strongly expressing when positive. These data and our recently published findings (11) suggest that GLP-1RA drugs, such as semaglutide, may cause unwanted MAPK activation and growth of GLP-1R-expressing NETs, similar to the endogenous GLP-1 ligand (5). Conversely, most NENs lack GLP-1R and should be unaffected by these agents. While GLP-1RAs are contraindicated for medullary thyroid carcinoma, we found 0 of 29 thyroid NETs expressed GLP-1R. Hence, in contrast to rodent studies (4), GLP-1RAs may have little effect on human thyroid NETs. It is clear that more preclinical research examining potential protumorigenic effects of GLP-1RAs and related incretin mimetics (e.g., tirzepatide) on GLP-1R-positive NETs, particularly dNETs and pNETs, is needed to better understand the safety of these drugs in NEN patients or other GLP-1R-expressing cancers (12, 13).

## Methods

### Collection, processing, and culturing of patient NET spheroids for drug testing

Surgically resected samples were collected under the University of Iowa IRB protocols #199911057 and #201708847. Written informed consent was obtained from all patients. Short-term NET spheroids were isolated from patient tumor samples and were grown in DMEM/F12 medium supplemented with 10% FBS, 1% PEN/STREP, 1% glutamine, 10 mM nicotinamide, and 10 μg/mL insulin, as previously described (14, 15). For drug testing experiments, dNET-951 and iNET-932 spheroids were washed with DPBS and grown in DMEM/F12 medium supplemented with 10% FBS, 1% PEN/STREP, 1% glutamine, and in the presence or absence of 100 nM of semaglutide for 2 weeks. Cell growth was determined using the AlamarBlue metabolic assay (14).

### NEN tissue microarrays, immunohistochemistry, and immunofluorescence

The NEN tissue microarrays (TMAs) were generated as part of the University of Iowa NET SPORE Biospecimen Core collection under IRB protocol #199911057. To address intratumoral heterogeneity in NEN samples, the TMAs were constructed using three different areas (1 mm punch samples) for each patient tumor. NEN TMAs were sectioned onto glass slides at 4 μm thickness, deparaffinized, rehydrated, heat-induced epitope retrieved, and immunohistochemically stained for GLP-1R (Abcam, USA; rabbit monoclonal EPR23507-57) at 1:400 dilution or for insulin (Dako, USA; polyclonal) at 1:5 dilution. Slides were counterstained with hematoxylin. IHC quantifications were reported as the average signal of the three punch samples per tumor.

Immunofluorescence staining was performed by fixing NEN cells with 4% paraformaldehyde for 10 min, followed by overnight incubation with primary antibodies to synaptophysin (Abcam #32127) at 1:600 dilution or GLP-1R (Abcam EPR23507-57) at 1:400 dilution. Cells were washed and incubated with anti-rabbit secondary antibodies conjugated with Alexa Fluor 488 (Jackson ImmunoResearch, USA; # 711-095-152) at 1:500 dilution for 1 h and fixed with mounting medium containing DAPI (4′,6-diamidino-2-phenylindole) nuclear stain. Immunofluorescence images were taken using a fluorescence microscope (Olympus, USA) at 400 ms exposure time.

### RNA extraction and quantitative PCR

NET spheroids were established from surgically resected patient tumors (three dNET, five iNET, and three pNET), harvested, flash frozen, and stored at −80°C before RNA isolation. Total RNA was isolated from samples using TRIzol and further purified using RNA isolation columns. RNA quality was determined using the NanoDrop 2000 (Thermo Fisher Scientific, USA; # ND-2000). 0.5–1 μg of total RNA samples with A260/A280 and A260/A230 ratios above 1.8 were used for complementary DNA synthesis (Lamda, USA; #209). Complementary DNA samples were used for quantitative PCR (qPCR) analyses using the following oligo sets and SYBR Green qPCR mix (Lamda # qMX-Green).

**Table:** 

Oligo name	Oligo sequence
f-18S	GAG ACT CTG GCA TGC TAA CTA G
r-18S	GGA CAT CTA AGG GCA TCA CAG
f-GLP1R	GACCTTCGATGAATACGCCTG
r-GLP1R	TCCTCGCACTCCGACAAGT
f-ISL-1	GCGGAGTGTAATCAGTATTTGGA
r-ISL-1	GCATTTGATCCCGTACAACCT
f-TPH1	ACGTCGAAAGTATTTTGCGGA
r-TPH1	ACGGTTCCCCAGGTCTTAATC

### Western blot

Patient-derived NET spheroids (dNET-924 and iNET-932) were washed with DPBS and cultured in DMEM/F12 with no FBS for 2 h before treatment with vehicle control or 100 nM semaglutide for 10 min. Cell pellets were harvested and lysed with LDS sample buffer (Thermo Fisher Scientific). Samples were boiled at 100°C for 10 min and run in a 4–20% gradient gel. Protein was transferred to a polyvinylidene difluoride membrane and probed for phosphorylated ERK1/2 (Cell Signaling Technology, USA; 4370S) or ERK1/2 (Cell Signaling Technology, 9102S) at 1:1,000 dilution.

## Supplementary materials



## Declaration of interest

The authors declare that there is no conflict of interest that could be perceived as prejudicing the impartiality of the work reported.

## Funding

This work was supported by the University of Iowa NET SPORE P50CA174521 and P50CA302572, HCCC NET SPORE, HCCC Oberley, and HCCC P30 CA086862 grants.

## Author contribution statement

AMB, PHE, DEQ, JSD, CHC, and JRH contributed to the experimental design of these studies. JRH and CHC provided surgical specimens. AMB, PHE, EA, SAH, RET, and JEM performed experiments. PHE, AMB, DEQ, JSD, JRH, and CHC analyzed data and wrote this manuscript.
