# Supplementary material for: Variable GLP-1 receptor expression across diverse neuroendocrine neoplasms: implications for incretin therapies
Source: Endocr Oncol. 2025 Nov 25;5(1):e250051. doi: 10.1530/EO-25-0051 (PMC12648204; doi:10.1530/EO-25-0051)
Supplement: Supplementary file 1 [file supplementary_materials.pdf]

Supplementary Table 1:

Clinical annotation and insulin IHC results in GLP-1R positive pNET samples.

GLP-1R+ pNET	GLP-1R H-Score	Insulin H-Score	Clinical Annotation*
pNET-1	285	0	Non-functional pancreatic NET
pNET-2	260	0	Insulinoma
pNET-3	170	0	Non-functional pancreatic NET
pNET-4	117	0	Non-functional pancreatic NET
pNET-5	117	150	Non-functional pancreatic NET
pNET-6	103	0	Liver metastasis of pancreatic NET with carcinoid symptoms due to serotonin expression
pNET-7	62	200	Insulinoma
pNET-8	27	0	MEN1; gastrin-expressing pancreatic NET
pNET-9	7	0	Primary pancreatic NET matched to pNET-6
pNET-10	5	15	MEN1; Non-functional pancreatic NET

* Insulinoma is a clinical diagnosis based on the presence of an insulin-producing (confirmed by serum testing) pancreatic NET with attributable symptoms (i.e., “Whipple’s triad”); insulinomas are usually but not always insulin IHC positive; pancreatic NETs may be insulin IHC-positive without manifesting symptoms (i.e., clinically non-functional pancreatic NET).

Supplementary Table 2:

Tumor information of NET spheroids used for qPCR experiments.

Sample ID	Tumor Type	Tumor Grade	Patient Age	Patient Sex
dNET-762	duodenal NET (Lymph node metastasis)	G2	56	M
dNET-924	duodenal NET (Liver metastasis)	G2	60	F
dNET-2244	duodenal NET (Lymph node metastasis)	G3	57	M
dNET-951	duodenal NET (Lymph node metastasis)	G1	70	F
iNET-916	ileal NET (Primary tumor)	G1	73	M
iNET-932	ileal NET (Lymph node metastasis)	G1	39	M
iNET-933	ileal NET (Primary tumor)	G1	75	M
iNET-610	ileal NET (Lymph node metastasis)	G1	59	M
iNET-612	ileal NET (Lymph node metastasis)	G2	68	F
pNET-914	pancreatic NET (Primary tumor)	G2	31	M
pNET-617	pancreatic NET (Liver metastasis)	G2	78	F
pNET-621	pancreatic NET (Liver metastasis)	G2	65	M
